# Caffeine Effect on Cognitive Function during a Stroop Task: fNIRS Study

**DOI:** 10.1155/2020/8833134

**Published:** 2020-11-21

**Authors:** Yafei Yuan, Guanghao Li, Haoran Ren, Wei Chen

**Affiliations:** Department of Electronic Engineering, Center for Intelligent Medical Electronics, Fudan University, 200433 Shanghai, China

## Abstract

Acting as a brain stimulant, coffee resulted in heightening alertness, keeping arousal, improving executive speed, maintaining vigilance, and promoting memory, which are associated with attention, mood, and cognitive function. Functional near-infrared spectroscopy (fNIRS) is a noninvasive optical method to monitor brain activity by measuring the absorption of the near-infrared light through the intact skull. This study is aimed at acquiring brain activation during executing task performance. The aim is to explore the effect of coffee on cognitive function by the fNIRS neuroimaging method, particularly on the prefrontal cortex regions. The behavioral experimental results on 31 healthy subjects with a Stroop task indicate that coffee can easily and effectively modulate the execute task performance by feedback information of the response time and accuracy rate. The findings of fNIRS showed that apparent hemodynamic changes were detected in the bilateral VLPFC regions and the brain activation regions varied with different coffee conditions.

## 1. Introduction

Caffeine, primarily obtained from coffee in daily life, is the most widely and frequently used psychoactive drug over worldwide [1–4]. Coffee is a highly-concentrated source of caffeine about 2% [5]. Coffee consumption is associated with some health benefits including reduced risk of cardiovascular disease (CVD) [6], lower incidence of diabetes mellitus [7, 8], less Alzheimer's disease (AD) [9, 10], and decreased death from inflammatory diseases [11, 12]. Acting as a brain stimulant, coffee resulted in heightening alertness, keeping arousal, improving executive speed, maintaining vigilance, and promoting memory, which are associated with attention, mood, and cognitive function [13–15]. The most profound function is improving cognition which involves human activities of daily living. The benefits of cognition from coffee consumption are typically correlated with caffeine that exerts its effects through nonselective antagonism of adenosine A_1_ receptors (A_1_R) and A_2A_ receptors (A_2A_R) [10, 16]. Studies indicate that caffeine absorption is from the gastrointestinal tract rapid and reaches 99% after about 45 min ingestion [17]. Due to hydrophobic properties, the caffeine allows its passage through all biological membranes, including the blood-brain barrier. Peak plasma caffeine concentrations appeared between 15 and 120 min after oral ingestion [18]. Moreover, caffeine half-life ranges from 3 to 5 hours in humans [19]. The majority of the research studies have focused on cognitive function and generally showed consistent findings [20]. However, with respect to studies on the higher-order complex brain function which relies on frontal lobe function, there is no consistent result. Such functions include the ability to plan, conceptualize, initiate, and sustain behavioral with aims [1]. The cognitive function constructs which are known as executive functioning are sensitive to the ingestion of caffeine. Hence, knowing the underlying interaction between caffeine and human cognition is valuable for human health and production.

The functional neuroimaging method has been more inclined to be adapted in cognitive neuroscience [21, 22], which explores the constituent parts of the human cognitive system might be and how the human cognition works with other mental processes [23]. As a new functional neuroimaging technique, the functional near-infrared spectroscopy (fNIRS) is the noninvasive optical method to monitor brain activity by measuring the absorption of the near-infrared light between 650 nm and 950 nm through the intact skull [24]. What is more, the absorption spectra of oxygenated hemoglobin (Oxy-Hb) and deoxygenated hemoglobin (Deoxy-Hb) are distinct in this region [25]. The concentrations of Oxy-Hb and Deoxy-Hb can be calculated separately using the modified Beer–Lambert law [26, 27]. Thus, Jöbsis first proposed the application of near-infrared spectroscopy (NIRS) in vivo to acquire the concentration changes of Oxy-Hb and Deoxy-Hb by the diffusely scattered light measurements [28].

Compared with other neuroimaging technique as positron emission tomography (PET), functional magnetic resonance imaging (fMRI), and magnetoencephalography (MEG), the fNIRS has many advantages [29]. The main advantage is the ability to directly measure the functional contrasts such as Oxy-Hb, Deoxy-Hb, and total hemoglobin with very high temporal resolution which enables the studies on the hemodynamic response to neural activation [24]. fNIRS is an inexpensive, relatively portable, and tolerant of motion artifacts neuroimaging method compare with other measurements which is completely noninvasive and could be used in daily life [22, 30]. The fNIRS is widely employed to investigate human cognition [31–33]. The frontal areas of the brain, specifically the prefrontal cortex (PFC), are associated with cognition [34–36]. The PFC regions carry out executive functions including higher-order cognitive functions which are essential for planning and executing complex motor control actions [37–39]. Hence, in order to investigate the underlying mechanism relation between light and cognition, selecting the PFC as the region of interest (ROI) is a reasonable and effective research route method [40]. Furthermore, studies about how the coffee modulates the cognition is seldom reported which is important for human health.

In this work, the aim is to explore the effect of coffee on cognitive function by fNIRS neuroimaging method, particularly on PFC regions. As focusing on how the coffee affects the cognitive function, 31 participants were arranged to ingest coffee and carried on the Stroop task before and after coffee ingestion. Moreover, the variation in ROIs with modulation by coffee is inspected by fNIRS.

## 2. Materials and Methods

### 2.1. Participants

Thirty-one right-handed healthy participants (15 males and 16 females) were selected for the study purpose from the age group of 21 to 24 years. All participants had normal or corrected-to-normal vision and normal color vision. No participants had a history of neurological or psychiatric disorder and were under any psychotropic medication. The research experiment strictly followed the ethics regulation of Fudan University and obtained the consent of each participant.

### 2.2. Procedures and Materials

**Figure 1:**
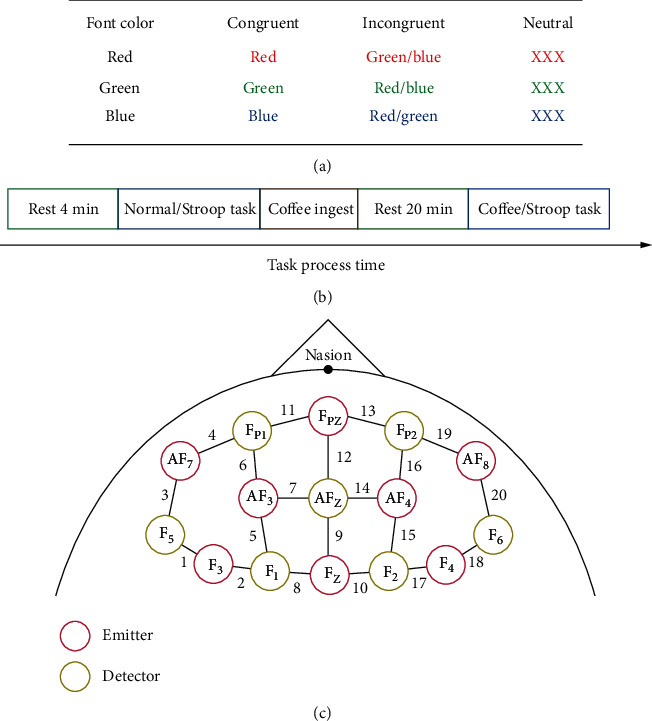
Task procedures and materials. (a) The color-word matching Stroop task rule. (b) The task process time. (c) The setting up of fNIRS channels.

All participants performed three Stroop tasks under three different light conditions. The modified color-word matching Stroop task was used as an event-related cognition task in this research [41, 42]. The functional neuroimaging method used the Stroop task for exploring human cognition [38, 42–44]. The rule of the single trial for the congruent, incongruent, and neutral conditions of color-word matching Stroop task is depicted in Figure 1(a). The single trial consisted of 12 words that were printed in black ink and appeared randomly. The complete task included 11 trials performed in one light mode. Participants were instructed to press the relevant response key during the executive task by the designed rules. The correct response action was to press the number keys “1,” “2,” and “3” for red, green, and blue color words, respectively. The response time and the response were recorded using a computer for further analysis.

The coffee effect task procedure is designed with two Stroop cycles as shown in Figure 1(b). Participants first performed the practice experiment and then carried out an actual procedure with over 92% practice accuracy rate. Participants intake a cup of Nescafe coffee about 210 ml after normal/Stroop task. The task process time is arranged by rest4minutes → normal/Strooptask → rest20minutes → coffee/Strooptask → end.

### 2.3. Data Acquisition by fNIRS

The fNIRS data were recorded using a multichannel continuous-wave fNIRS system instrument (NIRx Medical Technologies LLC-NIR-Scout, USA), which consists of eight LED light sources and eight photodetectors [38]. The distance between the detector and source is approximately 3 cm. The detector record relative changes in Oxy-Hb and Deoxy-Hb at a sample rate of 7.81 Hz at two wavelengths (760 and 850 nm) [45]. The location of probe placement and arrangement of specific brain regions was according to previous studies [46]. In this study, we focused on the PFC region for investigating cognition.  Figure 1(c) shows the set-up of fNIRS channels. Four different ROIs were selected: the right ventrolateral prefrontal cortex (R-VLPFC) (channels 17-20), the right dorsolateral prefrontal cortex (R-DLPFC) (channels 13-16), the left L-DLPFC (channels 5-7 and 11), and the posterior left L-VLPFC (channels 1-4). The fNIRS transmitters were specially designed to wrap with a tightly black bandage to ensure that there was no extraneous light interference during the Stroop cognitive task.

Participants were instructed to sit comfortably in a chair and maintain a calm and relaxed position. They were asked to focus on the screen with their minds blank. The visual of the task was presented on a 21-inch thin-film transistor (TFT) screen.

### 2.4. Data Processing

The fNIRS raw data analysis was executed based on SPM with additional modules for ANOVAs. First, a low-frequency band-pass filter (0.01-0.2 Hz) was applied to eliminate the baseline drift, artifact, and physiological noise. fNIRS records the changes in Oxy-Hb and Deoxy-Hb concentration simultaneously. However, there are some scientific problems in the selection of signals to analyze brain activation. In this research, it mainly focused on the Oxy-Hb signal changes, as it was normally observed to own higher amplitude than the Deoxy-Hb signal [45]. Furthermore, the signal-to-noise (S/N) ratio of Oxy-Hb is better, and the signal is more sensitive to process task response [47]. To explore whether those channels activated during the Stroop task, we analyzed channel-by-channel. If the light intensity of channels fell below 400 mV or exceeded 4,000 mV at any point during the session, then they were rejected [35]. The behavioral performance and Oxy-Hb data were analyzed in Vision 22.0 SPSS using a 3(stimulusconditions) × 2(coffeecondition) repeated-measure ANOVAs.

## 3. Results

### 3.1. Behavioral Results: Stroop Interference

**Figure 2:**
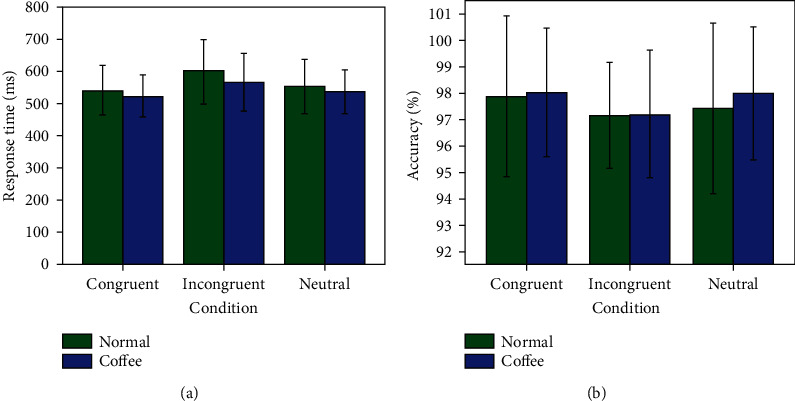
The results of Stroop task performance. (a) Response time with two conditions. (b) Accuracy rate with two conditions.

The results of Stroop task performance are shown in Table 1, including the response time and accuracy rate.  Figure 2 depicts the behavioral response time (RT) and accuracy rate results. Concerning the response time shown in Figure 2(a), by using the repeated-measure stimuluscondition(congruentvs.incongruentvs.neutral) × coffeecondition(normalvs.coffee), ANOVA demonstrated a significant effect for stimulus condition (*F* = 28.81, df = 2, *P* < 0.0005) and coffee conditions (*F* = 10.25, df = 1, *P* = 0.003). No significant interaction effect of the stimuluscondition × coffeecondition was observed (*F* = 2.13, df = 2, *P* = 0.128). Three stimulus condition under normal state (*F* = 26.52, df = 2, *P* < 0.0005) and coffee state (*F* = 13.76, df = 2, *P* < 0.0005) showed an obvious Stroop interference. Further, with regard to the coffee condition with different stimulus condition, the congruent (*F* = 5.10, df = 1, *P* = 0.031) and incongruent (*F* = 13.82, df = 1, *P* = 0.001) show significant difference, and neutral (*F* = 2.93, df = 1, *P* = 0.097) shows marginal significant difference. The RT of the incongruent stimulus condition was longer than the congruent and neutral stimulus condition in all normal and coffee states. Participants responded quickly after coffee ingestion condition than normal condition.

With respect to the accuracy rate shown in Figure 2(b), by using the repeated-measure stimuluscondition(congruentvs.incongruentvs.neutral) × coffeecondition(normalvs.coffee), ANOVA results demonstrated a marginally significant effect for stimulus condition (*F* = 2.45, df = 2, *P* = 0.095) and no significant effect for coffee condition. No main significant interaction effect of the stimuluscondition × coffeeconditions was observed. It is clear that the accuracy rate is higher under the coffee ingestion condition compared with normal condition in Figure 2(b). Furthermore, the accuracy rates were significantly lower in the incongruent condition compared with the congruent and neutral ones. As the behavioral results, it is clearly shown that the participants exhibit the best behavioral performance under the coffee ingestion condition than normal condition, not only due to faster response time but also more accuracy rate. In this research, coffee ingestion is regarded as the high-efficiency method to improve human performance.

### 3.2. fNIRS Results: Brain Activation

**Figure 3:**
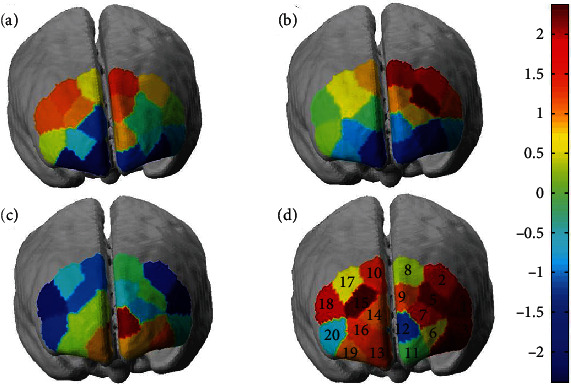
The brain activation under different conditions. (a) The brain activation under normal/rest condition. (b) The brain activation under normal/Stroop condition. (c) The brain activation under coffee/rest condition. (d) The brain activation under coffee/Stroop condition.

The brain activation of the *t*-statistic map for Oxy-Hb is shown in Figure 3 under four conditions which were normal/rest, normal/Stroop, coffee/rest, and coffee/Stroop. Figure 3(a) under normal/rest condition showed that there were no channels with brain activation of the Oxy-Hb level. Figure 3(b) under normal/Stroop condition showed brain activation of Oxy-Hb level in several channels (1, 2, 5, and 8) mainly locating at L-VLPFC. Figure 3(c) under coffee/rest condition showed brain activation of Oxy-Hb level in several channels (4, 6, 11, 12, and 13) mainly locating at L-VLPFC and L-DLPFC. Figure 3(d) under the coffee/Stroop condition showed brain activation of Oxy-Hb level in several channels (1, 2, 3, 4, 5, 7, 9, 10, 15, and 18) mainly locating at VLPFC. It is clear to see that the significantly different exhibited brain activation in PFC under normal vs. coffee conditions. The lowest mean Oxy-Hb level happened in normal/rest condition. While in normal/Stroop condition, it is required that a high mean Oxy-Hb level completes the task resulting in activating the channels (1, 2, 5, and 8). Furthermore, channel 12 was significantly activated under coffee/rest condition demonstrating the strong correlation between coffee effects in L-DLPFC. Moreover, compared with the Stroop task modes, it is found that the relevant ROIs change following the coffee intake. Compared to the normal/Stroop condition, more channels were involved in completing tasks under coffee/Stroop mainly located in R-VLPFC and L-VLPFC.

**Figure 4:**
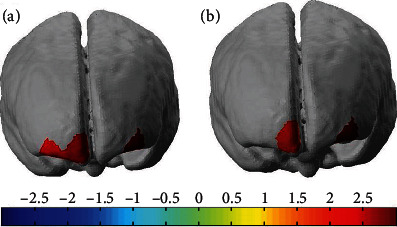
The significant difference in Oxy-Hb levels of the channels compared. (a) The comparison of Oxy-Hb levels with coffee/rest vs. normal/rest. (b) The comparison of Oxy-Hb levels with coffee/Stroop vs. normal/Stroop.

**Figure 5:**
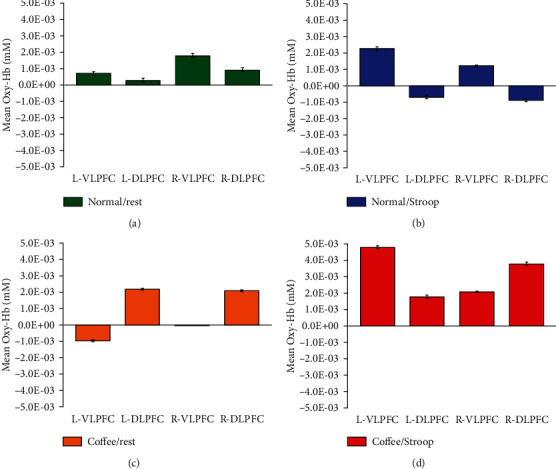
Mean Oxy-Hb concentration in PFC regions. (a) Mean Oxy-Hb concentration in PFC regions under normal/rest condition. (b) Mean Oxy-Hb concentration in PFC regions under normal/Stroop condition. (c) Mean Oxy-Hb concentration in PFC regions under coffee/rest condition. (d) Mean Oxy-Hb concentration in PFC regions under coffee/Stroop condition.

In order to locate the exact channel variates, we used a paired *t*-test to statistically compare the PFC activation under the coffee condition. The results of all channels with significant differences (*P* < 0.05) are shown in Figure 4. Figure 4(a) shows that the channels (4, 13, and 19) present significant differences between the normal/rest and normal/Stroop conditions. And Figure 4(b) shows that the channels (4 and 13) present significant differences between coffee/rest and coffee/Stroop conditions. In order to investigate the variate and hemodynamic response accompanying with getting worse light condition, we focus on the mean Oxy-Hb level of these ROIs such as L-VLPFC, L-DLPFC, R-VLPFC, and R-DLPFC.  Figure 5 illustrates the mean Oxy-Hb concentrations in PFC regions under four conditions. Significant differences were observed in normal and coffee conditions. It is clear to see the apparent changes in ROIs between the different coffee conditions. The mean Oxy-Hb concentration in L-VLPFC, L-DLPFC, R-VLPFC, and R-DLPFC increases with coffee intake. Under rest conditions, the coffee intake makes Oxy-Hb concentration of the L-DLPFC and R-VLPFC regions significantly rising. Under Stroop task conditions, the coffee intake makes Oxy-Hb concentration of the L-VLPFC, L-DLPFC, and R-DLPFC regions significantly rising.

## 4. Discussion

**Figure 6:**
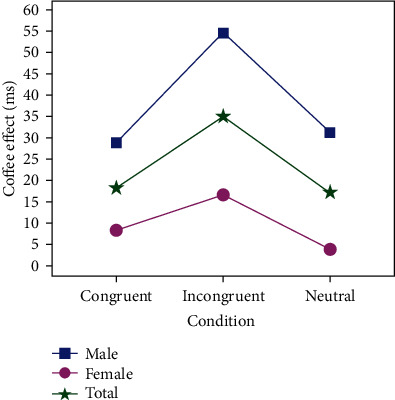
The results of the coffee effect on behavioral performance.

The present study is aimed at investigating how coffee intake on the cognitive function and PFC brain activation effect during the color-word matching Stroop task. In this research, the Stroop interference results existed in normal and coffee conditions were in accordance with the literature [41]. The results indicated that coffee condition could improve the participant's behavioral performance during executive tasks while comparing the normal condition which is consistent with work [20]. The findings were concluded not only based on the response time results but also the accuracy rate results. Participants under the coffee/Stroop condition perform faster executive speed with a higher accuracy rate compared to under normal/Stroop. That is to say, people could keep efficient working by coffee intake condition. As shown in Figure 6, it depicted the coffee effect on behavioral performance by using coffee/Stroop condition subtracting normal/Stroop concerning to response time. Clearly, the coffee condition is higher than the normal condition in all kinds of stimulus condition, which have more facilitative effects on the executive functions resulting in fast response and high accuracy. Specially, coffee could improve more performance in the complex task (incongruent) compared to easy task (congruent and neutral). Interestingly, there is some significant difference between males and females on the coffee effect under all stimuli. The coffee takes a stronger effect on behavioral performance with males compared to females, which proves males are more sensitive to coffee.

**Figure 7:**
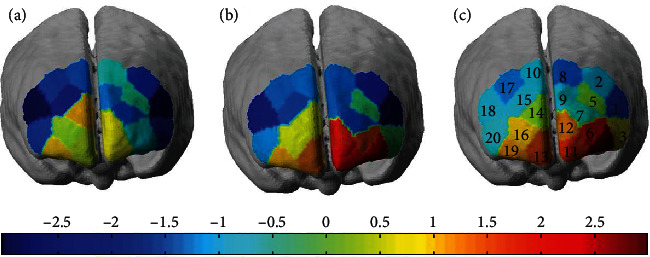
The brain activation under coffee/rest condition. (a) The brain activation after coffee ingestion 10 min. (b) The brain activation after coffee ingestion 15 min. (c) The brain activation after coffee ingestion 20 min.

Additionally, the neuroimaging results by fNIRS demonstrated the Oxy-Hb levels in response to the Stroop task in the bilateral VLPFC and DLPFC. The apparent hemodynamic changes were detected in the bilateral VLPFC regions under different conditions which are consistent with previous studies on cognition by event-related neuroimaging [38, 43, 44]. Under the efficiency work light condition related with coffee ingestion, the VLPFC was activated by mainly increasing several channel Oxy-Hb levels (1, 2, 3, 4, 7, 10, 15, and 18) mainly located in L-VLPFC. The VLPFC started to be activated due to coffee ingestion to execute tasks. Previous studies concluded that the activation of the right DLPFC may be due to the sensitivity in task difficulty [48]. A similar phenomenon was observed in our study, as the Oxy-Hb level of DLPFC increases under the coffee/Stroop condition shown in Figure 6(d). In order to explore the process of coffee effect, Figure 7 shows the process of coffee ingestion within 20 min, which clearly describes the process of variations in PFC. The mean Oxy-Hb level of channels (4 and 6) increase after coffee ingestion.

According to the behavioral performance and fNIRS results, it was concluded that coffee intake could effectively improve human cognitive function. It was proposed that humans show better performance with accomplishing high-efficiency work within coffee condition. This research data demonstrates that coffee intake condition makes people efficiently and easily undergoes the mission. In summary, this study confirmed that coffee can be regarded as a safe, effective, less expensive, and accessible tool to modulate human cognitive function. Additionally, the fNIRS is a reliable method to explore the mechanisms of brain activation for cognitive performance in relation to relevant factors.

## 5. Conclusion

In conclusion, the present observations confirm that the fNIRS is a noninvasive, less expensive, and accessible neuroimaging method. It is well-suited for monitoring PFC changes associated with cognitive functions. This study has focused on the effect of coffee intake on human cognition and explored the underlying mechanism. The behavioral results indicate that coffee can easily and effectively modulate the execution task performance by the means of feedback information, such as response time and accuracy. The findings of fNIRS show that the apparent hemodynamic changes were detected in the bilateral VLPFC regions, and the brain activation regions variate with different coffee condition. Furthermore, it reveals that coffee is a sensitive modulator for cortical hemodynamic correlated with cognitive function. By combining the two results, human behavioral performance benefit from coffee intake and show high-efficiency work. Moreover, coffee could be regarded as a safe, effective, inexpensive, and accessible method to modulate human cognitive function, which is of significant importance for human daily life.

## Figures and Tables

**Table 1:** Stroop task performance results.

	Response time (ms)			Accuracy (%)		
	Congruent	Incongruent	Neutral	Congruent	Incongruent	Neutral
Normal	541.5 ± 76.9	601.4 ± 103.0	553.9 ± 84.0	97.9 ± 3.1	97.2 ± 2.0	97.4 ± 3.2
Coffee	523.3 ± 66.0	566.5 ± 89.2	536.7 ± 84.1	98.0 ± 2.4	97.2 ± 2.4	98.0 ± 2.5

## Data Availability

The data used to support the findings of this study are available from the corresponding author upon request.
